# The association between physical activity and musculoskeletal disorders—a cross-sectional study of teachers

**DOI:** 10.7717/peerj.14872

**Published:** 2023-02-22

**Authors:** Małgorzata Grabara

**Affiliations:** Institute of Sport Science, Jerzy Kukuczka Academy of Physical Education, Katowice, Poland

**Keywords:** Seven-Day Physical Activity Recall, Nordic Musculoskeletal Questionnaire, Neck pain, Low back pain

## Abstract

**Purpose:**

Combined with insufficient physical activity (PA) prolonged and improperly performed sedentary work can lead to musculoskeletal disorders (MSDs). The aim of this study was (I) to evaluate the self-reported level of PA and the prevalence of MSDs in male and female teachers, and (II) to investigate the association between PA and MSDs in teachers in Polish primary and secondary schools.

**Methods:**

The study included 254 teachers from primary and secondary schools from Upper Silesia, Poland, excluding physical education teachers. The level of PA was assessed using the Seven-Day Physical Activity Recall (SDPAR). A standardized Nordic Musculoskeletal Questionnaire (NMQ) was used to assess the prevalence of MSDs.

**Results:**

A similar percentage of female (80%) and male (90%) teachers met the WHO recommendations on moderate-intensity PA. The recommendations on performing vigorous-intensity PA were met by significantly (*p* = 0.002) less female than male teachers (50% and 75% respectively). Lower back disorders during the last 12 months and the last 7 days (57% and 45%, respectively) were the most commonly reported MSDs by teachers, followed by neck (53%, 40%), upper back (39%, 28%), and knee disorders (37%, 26%). The highest pain intensity was experienced by the teachers in the lower back and neck. Teachers with a greater number of MSDs were less likely to engage in vigorous-intensity PA and total PA than those with fewer painful areas of the body. Pain intensity in the neck, knees, upper- and lower back, and wrists/hands was negatively related to moderate and total PA. BMI negatively correlated with total PA, moderate-intensity PA vigorous-intensity PA, and high vigorous-intensity PA.

**Conclusions:**

The study revealed the association between PA and MSDs in studied teachers. The most of the studied teachers met the WHO recommendation, and women were less likely to perform vigorous and high-vigorous PA than men. The lower back and neck disorders were the most common among the teachers.

## Introduction

Previous studies have identified musculoskeletal disorders (MSDs) as one of the greatest public health problems worldwide and a frequent cause of absenteeism in the workplace (Matikainen & Sjögren, 2020; Pan et al., 2019; Roman-Liu, 2008). MSDs are injuries or dysfunctions affecting muscles, ligaments, joints, bones, nerves, and spinal discs. The most common symptoms of MSDs are pain, joint stiffness, tingling and numbness in the muscles, as well as reduced mobility and functional impairment. They can be divided according to duration (acute and chronic), localization (local and general), and causes (primary and secondary) (Da Costa & Vieira, 2010; Malińska, 2019). According to the Social Insurance Institution (ZUS), MSDs are the third most common cause of incapacity for work in Poland (Bugajska, Żołnierczyk-Zreda & Hildt-Ciupińska, 2011). Both excessive physical workload and prolonged and improperly performed sedentary work can cause MSDs. The risk factors of MSDs include insufficient physical activity, lifting and carrying loads, computer work, static loads, awkward and/or sustained postures, abnormal sagittal spinal curvatures, prolonged sitting and standing, as well as being overweight and smoking (Da Costa & Vieira, 2010; Malińska, 2019; Zwierzchowska & Tuz, 2018; Gaweł & Zwierzchowska, 2021; Zwierzchowska et al., 2022). However, each type of MSDs (*e.g*., lower back pain, knee, or hand/wrist pain) has different risk factors (Da Costa & Vieira, 2010; Malińska, 2019).

The effects of physical activity (PA), as primary and secondary prevention of lifestyle diseases, *e.g*. cardiovascular or metabolic diseases, have been well documented in previous studies (Garber et al., 2011; Petermann-Rocha et al., 2019; Reiner et al., 2013; Sanz, Gautier & Hanaire, 2010). Participation in regular PA can help meet work requirements and enhance the quality of life regardless of age (Koolhaas et al., 2018; Grabara, Nawrocka & Powerska-Didkowska, 2018). Previous studies have revealed a significant positive relationship between PA and the ability to work (Grabara, Nawrocka & Powerska-Didkowska, 2018; Arvidson et al., 2013; Nawrocka et al., 2019). High levels of PA during leisure time have been associated with a lowered risk of long-term sickness absence (López-Bueno et al., 2020). Furthermore, leisure-time PA can prevent and reduce the occurrence of MSDs in all age groups (Nawrocka et al., 2019; Buyukavci, Akturk & Akturk, 2020; Nawrocka et al., 2014; Murata et al., 2019). On the other hand, high intensity and volume of PA can increase the risk of injury and contribute to the occurrence of MSDs (Kesaniemi et al., 2001; Grabara & Sadowska-Krępa, 2021). Similarly, certain types of occupational activity are not beneficial due to insufficient intensity, duration, and static postures (Hallman, Jørgensen & Holtermann, 2017). Previous studies focusing on the association between different domains of PA and MSDs have shown inconsistent findings (Hoogendoorn et al., 1999; Hildebrandt et al., 2000).

Engaging in PA is particularly important among white-collar workers (*e.g*., teachers), for whom the main body position at work is sedentary. Combined with insufficient PA, prolonged and improperly performed sedentary work can lead to MSDs. The teaching profession is characterized by a high mental workload, high levels of stress and physical complaints, low job satisfaction, and relatively high absenteeism (Grabara, Nawrocka & Powerska-Didkowska, 2018; Maguire & O’Connell, 2007). Previous studies have shown that the risk of developing MSDs also depends on psychosocial factors such as time pressure, low job satisfaction, high demands, and insufficient social support (Bongers, Kremer & Ter, 2002; Hoogendoorn et al., 2000; Häkkänen, Viikari-Juntura & Martikainen, 2001; Bugajska et al., 2011). Therefore, it is important to promote PA among this group of employees.

The aim of this study was (I) to evaluate the self-reported level of PA and the prevalence of MSDs among male and female teachers, and (II) to investigate the association between PA and MSDs in teachers in Polish primary and secondary schools. It was hypothesized that men would be characterized by higher levels of PA than women, the most commonly reported MSDs would be lower back pain, and the prevalence of MSDs would be related to the PA.

## Materials and Methods

### Participants

The study population consisted of teachers (excluding physical education teachers) from public primary and secondary schools in Upper Silesia, Poland. The sample included all teachers from randomly selected schools that met the inclusion criteria. The randomization was based on drawing 18 districts/cities with district rights from 36 districts/cities with district rights from Upper Silesia, and then drawing one school in a given district/city. The total number of invited teachers from the drawn schools was 590 (excluding physical education teachers), some of whom did not agree to participate in the study or did not meet the other inclusion criteria. Eventually, 254 teachers participated in the final analysis. The inclusion criteria were at least 1 year of working as a teacher, no contraindications to PA, and consent to participate in the study. The exclusion criteria were pre-existing injuries, illnesses, or diseases that restrict the ability to engage in PA.

### Methods and procedures

This study was approved by the Bioethics Committee of the Jerzy Kukuczka Academy of Physical Education in Katowice (certificate of approval No. KB/02/12) and conformed to the standards set by the Declaration of Helsinki. All participants were informed about the procedure and purpose of the study and gave their informed consent prior to filling out the questionnaire. The study was conducted using a direct pen-and-paper interview method in the workplace of the studied teachers.

The level of PA was assessed using the Stanford Seven-Day Physical Activity Recall (SDPAR). SDPAR is a valid measure for monitoring PA in healthy adults (Gross et al., 1990). However, body mass, percent fat, BMI, and obesity type may affect the SDPAR score (Lipert & Jegier, 2017; Washburn et al., 2003). SDPAR is also considered a useful tool for the assessment of PA in preventive screening for cardiovascular diseases (Czeczelewska et al., 2016). The SDPAR is conducted by an interviewer and is widely used in epidemiological studies (Blair et al., 1985; Garfield et al., 2012). Teachers were asked to estimate the number of minutes spent in the last 7 days for moderate-intensity PA (MPA, described as 3.0–5.0 METs), vigorous-intensity PA (VPA, described as 5.1–6.9 METs), high vigorous-intensity PA (HVPA, described as ≥7.0 METs) (Blair et al., 1985). The teachers were asked to indicate only PA lasting for at least 10 min. To convert the declared time of PA in minutes to METmin, a value of 4 METs was adopted for MPA, a value of 6 METs for VPA, and a value of 10 METs for HVPA (Blair et al., 1985). Taking all the data into consideration, the total energy expenditure of PA per day and week (kcal per day, kcal per week) was calculated (Blair et al., 1985; Gross et al., 1990; Kaleta, Makowiec-Da̧browska & Jegier, 2004; Grabara & Sadowska-Krępa, 2021).

Data collected from SDPAR allowed the identification of the studied teachers who fulfilled the recommendation of the World Health Organization (WHO) regarding aerobic activity. According to this recommendation, for substantial health benefits, adults aged 18 to 65 should complete at least 150–300 min a week of aerobic MPA, or 75–150 min of aerobic VPA, or an equivalent combination of aerobic MPA and VPA throughout the week. Additional health benefits can be achieved by performing aerobic MPA for more than 300 min, or by performing aerobic VPA more than 150 min, or performing an equivalent combination of aerobic MPA and VPA throughout the week (WHO, 2020).

A standardized Nordic Musculoskeletal Questionnaire (NMQ) was used to assess the prevalence of MSDs. Previous studies have concluded that the NMQ is repeatable, sensitive, and useful as a screening and surveillance tool, and it has been applied to a wide range of occupational groups to evaluate MSDs (Dickinson et al., 1992; Palmer et al., 1999; Crawford, 2007). The NMQ contains a special map of the back of the human body separated into the nine anatomical localizations: neck, shoulders, upper back, elbows, lower back, wrists/hands, hips/thighs, knees, and ankles/feet. The teachers were asked if they had had any musculoskeletal problems (discomfort, numbness, or ache) in each of these areas during the past 12 months and the past 7 days. In the case of pain complaints over the past 7 days, pain intensity was assessed on a scale of 1 to 10 (from minimal to intense to unbearable pain) (Nawrocka et al., 2014; Grabara & Sadowska-Krępa, 2021; Kuorinka et al., 1987).

Body height and body mass were self-reported by the teachers and used to calculate their body mass index (BMI, kg/m^2^).

### Statistical analysis

The results are expressed as means and standard deviations (M ± SD), confidence intervals (−95% to 95%), or described using frequencies (percentage), and minimal and maximal values. The normality of distribution and homogeneity of variance were tested using the Shapiro–Wilk test and Levene’s test, respectively. Differences in PA between male and female teachers were analyzed using the Mann-Whitney U test. Between-group differences in meeting/not meeting WHO recommendation were analyzed using Pearson’s Chi-squared test.

The relationships between the prevalence of each MSD item during the last 12 months and the last 7 days, and the quantitative variables of PA (weekly PA level) were assessed using the Mann-Whitney U test. Pearson’s Chi-squared test was used for the qualitative variables of PA (defined as those which met or did not meet the WHO recommendation). The relationships between the sum of all MSDs during the last 12 months or during the last 7 days, pain intensity, age, body mass, BMI and the quantitative variables of PA were assessed using Spearman’s rank correlation. Spearman correlation coefficient rho was qualitatively evaluated as follows: up to 0.2 as trivial, 0.2 to <0.4 as weak, 0.4 to <0.6 as moderate, 0.6 to <0.8 as strong, 0.8 to 1 as very strong. The effect size was evaluated using the r index (Mann–Whitney U test): 0.1 to <0.3—small effect, 0.3 to <0.5—medium effect, ≥0.5—large effect, or Cramer’s V (Pearson’s Chi-squared test): 0.1 to <0.2—small effect, 0.2 to <0.4—moderate, 0.4 to <0.6—relatively strong, 0.6 to <0.8—strong, 0.8 to 1—very strong effect.

The level of significance in all tests was set as α = 0.05. The statistical analysis was performed using PS Imago Pro 7.0 software (IBM SPSS Statistics 27).

## Results

The participants’ demographics are presented in Table 1.

**Table 1:** Participants demographics.

Variables	*n*	%
Gender		
Men	51	20%
Women	203	80%
Age (years) M ± SD = 39.8 ± 9.28; min-max 24–66		
≤35	104	41%
36–50	111	44%
>50	39	15%
Body height (cm) M ± SD = 168.8 ± 8.31; min-max 150–198		
Body mass (kg) M ± SD = 71 ± 13.19; min-max 42–120		
BMI (kg/m^2^) M ± SD = 24.81 ± 3.65; min-max 17.94–37.78		
Underweight	5	2%
Healthy weight (norm)	136	54%
Overweight	90	35%
Obese	23	9%
Experience in the profession (years)		
<5	38	15%
5–9	68	27%
10–14 15–20	51 45	20% 18%
>20	42	20%

**Note:**

M ± SD, mean and standard deviation; BMI, body mass index.

**Figure 1:**
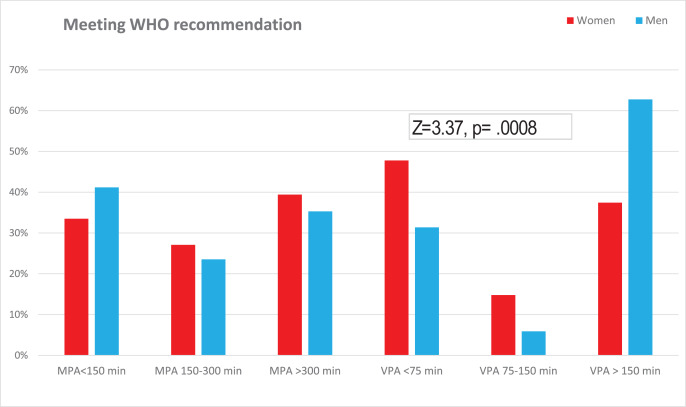
Meeting the WHO recommendations by studied teachers.

Most of the studied teachers, with a similar percentage of women and men (80% and 90%, respectively), declared their participation in MPA at least 150 min per week, whereas 50% of women and 75% of men declared their participation in VPA or/and HVPA at least 75 min per week, with this difference being statistically significant (Chi^2^ = 9.33, *p* = 0.002, V = 0.19). Meeting the WHO recommendations by studied teachers is presented in Fig. 1. It was found that male teachers were more likely to engage in VPA or/and HVPA than female teachers, and this difference was statistically significant (Z = 3.37, *p* = 0.0008 r = 0.21).

The self-reported levels of PA during the week, workweek, and weekend are presented in Table 2. Differences between women and men were found in VPA and HVPA, and total weekly energy expenditure on PA. However, the effect size was small (<0.30) (Table 2). The comparison of participation in PA during the workweek *vs*. on weekend did not reveal any statistically significant differences.

**Table 2:** Physical activity (PA), energy expenditure on PA of the participants, and differences in the level of PA between women and men.

Variables	Participants (*n* = 254)					
	Women (*n* = 203)		Men (*n* = 51)		Sig.	Effect size
	M ± SD	C.I	M ± SD	C.I.		
Weekly PA (minutes)						
Moderate-intensity (MPA)	599.3 ± 584.4	[518–680]	536.1 ± 454	[408–663]	0.969	
Vigorous-intensity (VPA)	105 ± 156.4	[83–126]	136.1 ± 154.2	[92–179]	**0.012**	**r = 0.16**
High-vigorous-intensity (HVPA)	33.2 ± 63.9	[24–42]	79.1 ± 108.5	[48–109]	**<0.001**	**r = 0.22**
Weekly PA (METmin)						
Moderate-intensity (MPA)	2,397 ± 2,337.6	[2,073.5–2,720.6]	2,144.3.5 ± 1,816.1	[1,633.5–2,655.1]	0.969	
Vigorous-intensity (VPA)	627.7 ± 938.5	[499.8–759.6]	816.5 ± 925.1	[556.3–1,076.7]	**0.012**	**r = 0.16**
High-vigorous-intensity (HVPA)	331.6 ± 639.3	[243.1–420.1]	791.2 ± 1,085.2	[485–1,096.4]	**<0.001**	**r = 0.22**
Total	3,358.3 ± 3,013.4	[2,941.3–3,775.4]	3,752 ± 2,731	[2,983.8–4,520.1]	0.108	
Monday to Friday PA (METmin)						
Moderate-intensity (MPA)	1,746.5 ± 1,829.8	[1,493.3–1,999.7]	1,588 ± 1,396.8	[1,195.4–1,981.1]	0.647	
Vigorous-intensity (VPA)	413.9 ± 696.4	[317.6–510.3]	586.5 ± 805.7	[359.9–813.1]	**0.029**	**r = 0.14**
High-vigorous-intensity (HVPA)	209.6 ± 445.3	[147–271.2]	500 ± 865.2	[256.7–743.3]	**0.004**	**r = 0.18**
Weekend PA (METmin)						
Moderate-intensity (MPA)	650.5 ± 598.9	[567.7–733.4]	556.1 ± 505.3	[413–698.2]	0.419	
Vigorous-intensity (VPA)	215.8 ± 312	[172.6–258.9]	230 ± 323.5	[139–320]	0.781	
High-vigorous-intensity (HVPA)	122 ± 266	[85.2–158.8]	291.2 ± 372.5	[186.4–395.9]	**<0.001**	**r = 0.22**
Weekly energy expenditure on PA (kcal)						
Total	3,587.4 ± 3,174.3	[3,148.1–4,026.7]	5,413.5 ± 4,072.7	[4,268–6,558.9]	**<0.001**	**r = 0.22**
Per day	512.5 ± 453.5	[449.7–575.2]	773.4 ± 581.8	[609.7–936]	**<0.001**	**r = 0.22**

**Note:**

C.I., confidence interval [–95% to 95%]; Sig., significance (*p*-values) calculated using *Mann-Whitney* U test; statistically significant values are bolded, r index: 0.1 to <0.3—small effect, 0.3 to <0.5—medium effect, ≥0.5—large effect.

The prevalence of MSDs during the last 12 months and the last 7 days is presented in Table 3. The 12-month prevalence of MSDs was 57% for lower back pain, 53% for neck pain, 38% for upper back pain, and 37% for knee pain. The remaining 7-day prevalence of MSDs in certain areas of the body was lower than in the last 12 months. The highest average pain intensity was reported for the lower back and neck (Table 3).

**Table 3:** Prevalence of musculoskeletal disorders (MSDs) during the last 12 months and the last 7 days among participants and pain intensity of MSDs during the last 7 days (*n* = 254).

Area of body affected	Occurrence of MSDs during last 12 months (*n*, %)	Occurrence of MSDs during last 7 days (*n*, %)	Pain intensity (1–10) of MSDs during last 7 days (min-max)	Pain intensity (1–10) of MSDs during last 7 days (M ± SD)
Neck	134 (52.8%)	101 (39.8%)	1–10	4.63 ± 2.15
Shoulders	67 (26.4%)	41 (16.1%)	1–10	4.29 ± 2.1
Upper back	98 (38.6%)	71 (28%)	1–10	4.5 ± 2.05
Elbows	28 (11%)	18 (7.1%)	1–5	2.5 ± 1.54
Wrists/hands	58 (22.8%)	40 (15.7%)	1–8	3.58 ± 2.06
Lower back	145 (57.1%)	115 (45.3%)	1–10	5.09 ± 2.39
Hips/thighs	48 (18.9%)	34 (13.4%)	1–8	3.65 ± 2.12
Knees	95 (37.4%)	67 (26.4%)	1–10	4.39 ± 2.31
Ankles/feet	60 (23.6%)	41 (16.1%)	1–9	3.71 ± 2.11

The association between weekly PA and the prevalence of MSDs during the last 12 months, the last 7 days, and the pain intensity during the last 7 days is presented in Table 4. The analysis revealed that teachers who did not report any MSDs during the last 12 months, nor the last 7 days (as listed in Table 4), performed more PA, particularly VPA and total PA, than the teachers who reported MSDs. The pain intensity was negatively correlated with participation in PA, particularly MPA and total PA (Table 4). Correlation analysis also indicated the relationship between VPA and the sum of all MSDs during the last 12 months (Spearman rho = −0.17, *p* = 0.007), as well as the sum of all MSDs during the last 7 days (Spearman rho = −0.19, *p* = 0.003). These correlations show that teachers complaining of multiple MSDs may be less likely to engage in VPA than those who had fewer MSDs. However, the observed correlations were trivial.

**Table 4:** Prevalence of musculoskeletal disorders during the last 12 months, and the last 7 days and pain intensity during the last 7 days in relation to weekly physical activity.

Area of body affected	Weekly physical activity			
	Moderate-intensity (MPA)	Vigorous-intensity (VPA)	High-vigorous-intensity (HVPA)	Total
During the last 12 months				
Neck yes = 134; no = 120		*p* = 0.023 r = 0.14		*p* = 0.003 r = 0.19
Shoulders yes = 67; no = 187		*p* = 0.004 r = 0.18		*p* = 0.003 r = 0.19
Knees yes = 95; no = 159		*p* = 0.028 r = 0.14		*p* = 0.043 r = 0.13
Ankles/Feet yes = 60; no = 194		*p* = 0.035 r = 0.13		
During the last 7 days				
Neck yes = 101; no = 153		*p* = 0.012 r = 0.16		*p* = 0.024 r = 0.14
Wrists/hands yes = 40; no = 214		*p* = 0.044 r = 0.12		
Hips/thighs yes = 34; no = 220	*p* = 0.023 r = 0.14			
Knees yes = 67; no = 187		*p* = 0.031 r = 0.13		
Pain intensity				
Neck	rho = −0.42 *p* < 0.001		rho= −0.26 p = 0.009	rho = −0.40 *p* < 0.001
Upper back	rho = −0.26 *p* = 0.032			rho = −0.28 *p* = 0.019
Wrists/hands	rho = −0.44 *p* = 0.005			rho = −0.34 *p* = 0.035
Low back pain	rho = −0.25 *p* = 0.007			
Knee	rho = −0.30 *p* = 0.012			rho = −0.30 *p* = 0.014

**Note:**

*p*, *p*-values calculated using *Mann-Whitney U* test or *Spearman correlation*; r, r index: 0.1 to <0.3—small effect, 0.3 to <0.5—medium effect, ≥0.5—large effect; rho, Spearman rho correlation coefficient: <0.2—trivial, 0.2 to <0.4—weak, 0.4 to <0.6—moderate, 0.6 to <0.8—strong, 0.8 to 1—very strong; *p*-values, r-index, and rho are given only for statistically significant differences or statistically significant correlations.

The analysis of the prevalence of MSDs in certain parts of the body during the last 12 months reported by the teachers in relation to meeting/not meeting WHO recommendations showed several limitations in meeting the recommendation regarding MPA for the teachers self-reporting MSDs of the wrists/hands (Chi^2^ = 3.97, *p* = 0.046, V = 0.13), ankles/feet (Chi^2^ = 4.92, *p* = 0.027, V = 0.14), and meeting the recommendation regarding VPA for the teachers self-reporting MSDs of the shoulders (Chi^2^ = 5.37, *p* = 0.020, V = 0.15), and knees (Chi^2^ = 4.31, *p* = 0.038, V = 0.13). A similar analysis of the prevalence of MSDs during the last 7 days showed that only hips/thighs pain was more often associated with not meeting the WHO recommendation regarding MPA by teachers (Chi^2^ = 3.88, *p* = 0.049, V = 0.12). However, the effect size was small (<0.2).

The study did not reveal any significant correlations between body mass, BMI, and the prevalence of MSDs in certain parts of the body during the last 12 months and during the last 7 days. However, it was observed that age was positively correlated with wrist/hand pain (Spearman rho = 0.16, *p* = 0.01), and knees pain (Spearman rho = 0.16, *p* = 0.01) self-reported during the last 12 months, and with wrist/hand pain (Spearman rho = 0.17, *p* = 0.007), lower back pain (Spearman rho = 0.15, *p* = 0.018), and hip/thighs pain (Spearman rho = 0.20, *p* = 0.001) reported during the last 7 days.

The analysis between quantitative variables of PA and age, body mass and BMI showed that age negatively correlated with HVPA (Spearman rho = −0.16, *p* = 0.010), body mass negatively correlated with total PA (Spearman rho = −0.13, *p* = 0.040), and MPA (Spearman rho = −0.15, *p* = 0.021), and BMI negatively correlated with total PA (Spearman rho = −0.19, *p* = 0.002), MPA (Spearman rho = −0.13, *p* = 0.039), VPA (Spearman rho = −0.14, *p* = 0.026), and HVPA (Spearman rho = −0.20, *p* = 0.002). However, these correlations were trivial or weak.

## Discussion

This study assessed the level of PA, the prevalence of MSDs, and the association between PA and MSDs in male and female primary and secondary schools teachers.

Over 80% of the teachers met the WHO recommendations on MPA, whereas male teachers were significantly more likely to meet the WHO recommendations on VPA and/or HVPA than female teachers. The comparison of quantitative variables of PA showed that male teachers were more likely to declare engaging in VPA and HVPA than female teachers. In their study of teachers that assessed PA by using IPAQ, Grabara, Nawrocka & Powerska-Didkowska (2018) found that male teachers had higher levels of VPA and MPA than female teachers (Grabara, Nawrocka & Powerska-Didkowska, 2018). In a study by Biernat & Tomaszewski (2015), the authors reported that 62% of working residents of Warsaw aged 20–69 years met the recommendation on healthy PA, and women were more frequently insufficiently active compared to men. Other studies have also demonstrated gender differences in PA levels and reported higher mean scores for PA in men compared to women (Gerovasili et al., 2015; Gauthier et al., 2012).

Lower back pain was the most commonly reported MSD followed by neck, upper back, and knee disorders. The high pain intensity experienced by the teachers was also reported in the lower back and neck. These findings are similar to previous studies on MSDs as several studies have also indicated that lower back pain and/or neck pain are the most commonly reported by teachers (Vega-fernández, Olave & Lizana, 2022; Durmus & Ilhanli, 2012; Solis-Soto et al., 2017; Scopa et al., 2020; Almeida et al., 2021; Tami et al., 2021; Abdel-Salam et al., 2021). Vega-fernández, Olave & Lizana (2022) found that 91% of Chilean teachers had some form of MSDs during the last 12 months, with the most commonly reported complaints being lower back pain (60%) and neck pain (47%). In Turkish teachers, the most common MSDs were lower back pain (75%), neck pain (48%), and knee pain (31%) (Durmus & Ilhanli, 2012). Solis-Soto et al. (2017) investigated the prevalence of MSDs in individual parts of the body over 12 months among 86% of Bolivian teachers. The most common MSDs were neck pain (47%) and knee pain (38%), whereas lower back pain and upper back pain were reported by 33% and 36% of participants, respectively (Solis-Soto et al., 2017). Scopa et al. (2020) studied the prevalence of MSDs in 92% of Italian teachers. Almeida et al. (2021) noted that active professors of the Instituto Federal do Sertão Pernambucano reported having felt some type of musculoskeletal symptom in the last 7 days, mostly in the lower back (53%) and lower limbs (29%). Tami et al. (2021) found that the neck and lower back were reported by university teachers in Cameroon as the areas of the body most affected by MSDs during both the previous 7 days and 12 months. Abdel-Salam et al. (2021) indicated the lower back (68%), knees (59%), shoulders (48%), and neck (45%) as the area most affected by MSDs over a 12-month period among female secondary school teachers.

As has been shown, the lower back and neck were commonly reported by teachers to be the areas most affected by MSDs. Lower back pain and neck pain may be caused by the sedentary postures experienced during their work. Prolonged sitting can overload the passive elements of the spine, and tilting the head forward can cause excessive tension in the neck muscles, leading to pain (Søndergaard et al., 2010; Waongenngarm, Rajaratnam & Janwantanakul, 2015). Bontrup et al. (2019) found a small association between general sitting behavior and chronic lower back pain in sedentary office workers. The authors indicated that the causes of lower back pain were multifactorial (Bontrup et al., 2019). Psychological factors such as low levels of job control, high psychological demands, and work dissatisfaction may also be responsible for the MSDs self-reported by teachers. High mental stress and occupational stress may increase muscle tension, which can lead to muscle fatigue and reduce the ability to relax (Da Costa & Vieira, 2010; Bongers, Kremer & Ter, 2002). Abdel-Salam et al. (2021) indicated an age above 40, more than 10 years of teaching, and uncomfortable school furniture as factors of work-related MSDs in female teachers from Saudi Arabia. Ndonye, Matara & Muriithi (2019) pointed to age, working in a head-down posture, lack of back support in chairs, teaching for over 4 h while standing, and teaching for over 4 h while sitting as factors associated with work-related MSDs in primary school teachers (Ndonye, Matara & Muriithi, 2019). In this study, age was also found to be a factor associated with MSDs in certain parts of the body, *i.e*. wrists/hands, knees, lower back and hips/thighs.

The present study examined the association between PA and the prevalence of MSDs. The teachers who reported neck, shoulder, knee, ankle, or foot pain during the last 12 months and neck, wrist, hands, or knee pain during the last 7 days participated in lower levels of VPA than those who did not report these complaints. Further analysis showed that teachers with a greater number of MSDs had a lower propensity to engage in VPA and total PA than those with fewer painful areas of the body. These findings are in line with those reported by Pan et al. (2019), who concluded that multiple-site pain was consistently related to increased low-intensity PA and reduced MPA to VPA. Moreover, Pan et al. (2019) found that people with a greater number of painful musculoskeletal sites have reduced levels of PA compared to those experiencing pain at two or fewer sites. Tami et al. (2021) did not find any association between PA and MSDs for MSDs occurring during the previous 7 days and 12 months. However, the authors observed a higher prevalence of 7-day MSDs in teachers who were inactive compared to those who were active. This was especially notable for the neck, lower back and upper back (Tami et al., 2021). Based on a study including 3,492 physicians, AIOmar (2021) found that a higher level of PA may prevent MSDs, although this association ceased to exist in a multivariable-adjusted model. Ezzatvar et al. (2020) stated that 75 min/week or more of leisure-time vigorous PA was associated with lower levels of neck and shoulder pain among physical therapists, whereas moderate leisure-time PA did not reveal any significant relationship with MSDs. High pain intensity may also contribute to decreased levels of PA. The results showed that pain intensity in the neck, knees, upper and lower back, and wrists/hands was negatively related to MPA and total PA. Therefore, PA may be avoided because of the pain.

The main focus of this study was to find an association between engaging in PA and the prevalence of MSDs. However, other factors, such as age, body mass, and overweight/obesity, may also affect participation in PA. The present study revealed that older teachers may participate in a lower levels of HVPA than younger teachers. Moreover, overweight/obese teachers may be less likely to engage in PA than those with normal BMI.

Further research is needed in larger populations to determine the possible relationships between engaging in PA and the prevalence of MSDs, as well as other factors that may affect the level of PA.

### Strengths and limitations of the study

The present study was conducted among teachers, who are a specific group of employees for whom the sedentary position can be considered the main position at work. In the present study, the teachers were recruited from the Upper Silesia region of Poland therefore, these results should not be generalized to the entire population of Polish teachers. There are several limitations of this study. First, the amount of PA and pain were measured by questionnaires. The types of PA (*e.g*. resistance training, flexibility/stretching) were not assessed which may affect the findings. As has been shown in previous studies, self-reported PA was often overestimated (Wick et al., 2016; Hagstromer et al., 2010; Shephard, 2003), and the perceived intensity of PA may vary depending on individual fitness or obesity levels (Hagstromer et al., 2010; Hukkanen et al., 2018). It is also difficult to capture pain intensity, frequency, and pattern. Second, the study did not consider meeting the WHO recommendations regarding muscle-strength activity. Third, the study did not consider body composition.

## Conclusions

The study revealed the association between PA and MSDs. Teachers who reported a greater number of MSDs were less likely to engage in VPA and total PA than those with fewer painful areas of the body, and the prevalence of MSDs may be associated with decreased vigorous PA and total PA. Overweight- or obese teachers may be less likely to be physically active than those who had normal BMI.

Most of the teachers in the present study self-declared participation in MPA at least 150 min per week, and the lower back and neck were the areas commonly found by teachers as those most affected by MSDs.

## Supplemental Information

10.7717/peerj.14872/supp-1 Supplemental Information 1 Information and questionnaire for the participants (English).

10.7717/peerj.14872/supp-2 Supplemental Information 2 Information and questionnaire for the participants (Polish).

10.7717/peerj.14872/supp-3 Supplemental Information 3 Raw data.

## Additional Information and Declarations

### Data Availability

The following information was supplied regarding data availability: The raw data is available in the Supplemental Files.
