# Supplementary material for: The association between physical activity and musculoskeletal disorders—a cross-sectional study of teachers
Source: PeerJ. 2023 Feb 22;11:e14872. doi: 10.7717/peerj.14872 (PMC9961098; doi:10.7717/peerj.14872)
Supplement: Supplemental Information 1 [file peerj-11-14872-s001.docx]

**Information for the participants:**

Dear Sirs,

We are employees of the University of Physical Education in Katowice and we are undertaking a study to assess the level of physical activity as a component of everyday life. The questions concern your physical activity during a typical week of your life (7 days), subjective assessment of your ability to work and musculoskeletal complaints. We assure you of the anonymity of your answers, but we ask you to be honest. The results of the survey will only be used for scientific purposes.

Małgorzata Grabara, Institute of Sport Science

## Questionnaire

## Overall part (includes basic data on the participants)

Gender: Man Woman

Age:……….. Body height [in cm]:…………. Body mass [in kg]:……….

How long have you been working in your current occupation?

less than 5 years 5-9 years 10-14 years 15-20 years over 20 years

□ □ □ □ □

***SDPAR Questionnaire (polish ver.)***

## *The Nordic Musculoskeletal Questionnaire (polish ver.)*

I have used these questionnaires in accordance with a published license.

The questionnaire is available from the original paper by Kuorinka et al.^[[1]](#footnote-1)^ and from Evaluation of Human Work, a Practical Ergonomics Methodology^[[2]](#footnote-2)^.

1. Kuorinka I, Jonsson B, Kilbom A et al. Standardized Nordic questionnaires for the analysis of musculoskeletal symptoms. Appl Ergon 1987; 18:233–237. [↑](#footnote-ref-1)
2. Wilson JR, Corlett EN. Evaluation of Human Work: A Practical Ergonomics Methodology, 1st ed. London: Taylor and Francis, 1992; 563–568. [↑](#footnote-ref-2)
