# Supplementary material for: The association between physical activity and musculoskeletal disorders—a cross-sectional study of teachers
Source: PeerJ. 2023 Feb 22;11:e14872. doi: 10.7717/peerj.14872 (PMC9961098; doi:10.7717/peerj.14872)
Supplement: Supplemental Information 2 [file peerj-11-14872-s002.docx]

Szanowni Państwo,

Jesteśmy pracownikami Akademii Wychowania Fizycznego w Katowicach i podejmujemy badania oceny poziomu aktywności fizycznej będącej składnikiem życia codziennego. Pytania dotyczą Państwa aktywności fizycznej w ciągu typowego tygodnia życia (7 dni), subiektywnej oceny zdolności do pracy zawodowej oraz dolegliwości mięśniowo-szkieletowych. Zapewniamy państwu anonimowość wypowiedzi, jednak prosimy o szczere odpowiedzi. Wyniki badania zostaną wykorzystane wyłącznie do celów naukowych.

Małgorzata Grabara, Katedra Turystyki i Prozdrowotnej Aktywności Fizycznej

## CZĘŚĆ OGÓLNA

Zawiera podstawowe dane dotyczące badanych

Płeć: K M

Wiek:……….. Wysokość ciała (wzrost) [w cm]:…………. Masa ciała [w kg]:……….

Obwód pasa [w cm]: Obwód bioder [w cm]:

Jak długo pracuje Pan/Pani w aktualnie wykonywanym zawodzie

krócej niż 5 lat 5-9 lat 10-14 lat 15-20 lat powyżej 20 lat

□ □ □ □ □

Ile czasu w ciągu dnia spędza Pan/Pani siedząc?

do 2 godzin 2-4 godzin 4-6 godzin 6-8 godzin powyżej 8 godzin

w ciągu jednego

typowego dnia życia □ □ □ □ □

(łącznie: w pracy i

poza pracą)

w ciągu jednego

typowego dnia w □ □ □ □ □

pracy (tylko w pracy)

***Kwestionariusz SDPAR***

Kwestionariusz dotyczy aktywności fizycznej podejmowanej w ciągu 7 ostatnich dni. Proszę brać pod uwagę tylko wysiłki trwające co najmniej **10 minut.**

**Przykłady aktywności ruchowej:**

**O umiarkowanej intensywności:**

Praca zawodowa: marsz, dostarczenie korespondencji, malowanie wnętrz, podnoszenie i przenoszenie lekkich przedmiotów.

Prace domowe: wycieranie kurzu i podłogi, mycie okien, grabienie trawnika, zmywanie naczyń,

nakrywanie do stołu.

Aktywność sportowa (rzeczywisty czas ruchu): siatkówka, tenis stołowy, intensywny spacer (ok. 5 km/godz.) golf, gimnastyka.

**O dużej intensywności (ciężkie):**

Praca zawodowa: prace ciesielskie i stolarskie, prace budowlane.

Prace domowe: szorowanie podłóg.

Aktywność sportowa ( rzeczywisty czas ruchu): tenis (parami), taniec, spokojny jogging.

**O bardzo dużej intensywności ( bardzo ciężkie):**

Praca zawodowa: kopanie, przenoszenie ciężkich ładunków.

Aktywność sportowa (rzeczywisty czas ruchu): tenis (singiel), intensywne bieganie, pływanie, piłka nożna, koszykówka.

| Dni tygodnia | Sen (w godz. z dokładnością do 0.5 h) | Wysiłki o umiarkowanej intensywności (w minutach) | Wysiłki o dużej intensywności (w minutach) | Wysiłki o bardzo dużej intensywności (w minutach) |
| --- | --- | --- | --- | --- |
| Poniedziałek |  |  |  |  |
| Wtorek |  |  |  |  |
| Środa |  |  |  |  |
| Czwartek |  |  |  |  |
| Piątek |  |  |  |  |
| Sobota |  |  |  |  |
| Niedziela |  |  |  |  |

W porównaniu z aktywnością ruchową w ciągu ostatnich 3 miesięcy, ostatni tydzień charakteryzował się:

1. Większą aktywnością ruchową
2. Podobną aktywnością ruchową
3. Mniejszą aktywnością ruchową

W przypadku aktywności ruchowej, którą trudno jest zakwalifikować wyszczególnij poniżej:

Rodzaj aktywności( krótki opis) Dzień podejmowania i czas (minuty)

…………………………………………………..................... ……………………………………………………………

…………………………………………….…….................... ……………………………………………………………

…………………………………………………..................... ………………………………………………………..…

………………………………………………....…………………... ……………………………………………………………

## SUBIEKTYWNA OCENA ZDOLNOŚCI DO PRACY

Proszę podać czas aktywności fizycznej o co najmniej umiarkowanej intensywności,

podejmowanej w trakcie pracy zawodowej w ciągu dnia.

( wysiłki podczas pracy zawodowej prowadzące do przyspieszenia pracy serca i oddechu, uczucia ciepła, pocenia, zadyszki).

□ do 60 minut w ciągu dnia

□ do 120 minut w ciągu dnia

□ do 180 minut w ciągu dnia

□ powyżej 180 minut w ciągu dnia

1. Jak ocenia Pan/Pani swoją aktualną zdolność do pracy w porównaniu w z najlepszą w życiu

Proszę zaznaczyć na skali

0 1 2 3 4 5 6 7 8 9 10

Bardzo źle □ □ □ □ □ □ □ □ □ □ □ Bardzo dobrze

1. Jak ocenia Pan/Pani zdolność do pracy w odniesieniu do wymagań zawodu

Bardzo źle Źle Przeciętnie Dobrze Bardzo dobrze

1. Możliwości

sprostania wysiłkowi □ □ □ □ □

fizycznemu

1. Możliwości

sprostania trudnościom □ □ □ □ □

umysłowym

1. Liczbę schorzeń rozpoznanych u Pana/Pani przez lekarza ocenia Pan/Pani jako

(Proszę zaznaczyć na skali od 1 do 7)

1 2 3 4 5 6 7

Bardzo małą □ □ □ □ □ □ □ Bardzo dużą

1. Jak ocenia Pan/Pani upośledzenie zdolności do pracy z powodu schorzeń?

1 2 3 4 5 6

Bardzo małe □ □ □ □ □ □ Bardzo duże

1. Jak ocenia Pan/Pani ilość nieobecności w pracy w ostatnich 12 miesiącach

Proszę zaznaczyć na skali

1 2 3 4 5

Bardzo mało nieobecności □ □ □ □ □ Bardzo dużo nieobecności

1. Na podstawie prognozy jak ocenia Pan/Pani swoją zdolność do pracy na najbliższe 2 lata

Bardzo źle Średnio Bardzo dobrze

□ □ □

1. Jak ocenia Pan/Pani swoje zasoby psychiczne do pracy

1 2 3 4

Bardzo źle □ □ □ □ Bardzo dobrze

**Kwestionariusz dolegliwości mięśniowo-szkieletowych**

Odpowiedzi na pytania związane z nasileniem dolegliwości bólowych (kolumna 4; skala 1-10) wypełnić należy tylko w przypadku ich występowania w ciągu ostatnich **7 dni** (zaznaczenia odpowiedzi TAK w 3 kolumnie)

| **Czy wystąpiły u Pana/Pani dolegliwości bólowe określonych części ciała** | | | | | |
| --- | --- | --- | --- | --- | --- |
|  | W ciągu ostatnich  **12 miesięcy** | | W ciągu ostatnich  **7 dni** | | Ból w ciągu ostatnich 7 dni w skali 1-10  (1-ból minimalny;  10-ból nie do wytrzymania) |
| SZYJA/KARK | 🗌 NIE | 🗌TAK | 🗌 NIE | 🗌TAK | 🗌 🗌 🗌 🗌 🗌 🗌 🗌 🗌 🗌 🗌  1 2 3 4 5 6 7 8 9 10 |
| RAMIONA | 🗌 NIE | 🗌TAK | 🗌 NIE | 🗌TAK | 🗌 🗌 🗌 🗌 🗌 🗌 🗌 🗌 🗌 🗌  1 2 3 4 5 6 7 8 9 10 |
| GÓRNA CZĘŚĆ PLECÓW | 🗌 NIE | 🗌TAK | 🗌 NIE | 🗌TAK | 🗌 🗌 🗌 🗌 🗌 🗌 🗌 🗌 🗌 🗌  1 2 3 4 5 6 7 8 9 10 |
| ŁOKCIE | 🗌 NIE | 🗌TAK | 🗌 NIE | 🗌TAK | 🗌 🗌 🗌 🗌 🗌 🗌 🗌 🗌 🗌 🗌  1 2 3 4 5 6 7 8 9 10 |
| NADGARSTKI  /RĘCE | 🗌 NIE | 🗌TAK | 🗌 NIE | 🗌TAK | 🗌 🗌 🗌 🗌 🗌 🗌 🗌 🗌 🗌 🗌  1 2 3 4 5 6 7 8 9 10 |
| DOLNA CZĘŚĆ PLECÓW | 🗌 NIE | 🗌TAK | 🗌 NIE | 🗌TAK | 🗌 🗌 🗌 🗌 🗌 🗌 🗌 🗌 🗌 🗌  1 2 3 4 5 6 7 8 9 10 |
| BIODRA/UDA | 🗌 NIE | 🗌TAK | 🗌 NIE | 🗌TAK | 🗌 🗌 🗌 🗌 🗌 🗌 🗌 🗌 🗌 🗌  1 2 3 4 5 6 7 8 9 10 |
| KOLANA | 🗌 NIE | 🗌TAK | 🗌 NIE | 🗌TAK | 🗌 🗌 🗌 🗌 🗌 🗌 🗌 🗌 🗌 🗌  1 2 3 4 5 6 7 8 9 10 |
| KOSTKI/STOPY | 🗌 NIE | 🗌TAK | 🗌 NIE | 🗌TAK | 🗌 🗌 🗌 🗌 🗌 🗌 🗌 🗌 🗌 🗌  1 2 3 4 5 6 7 8 9 10 |

**
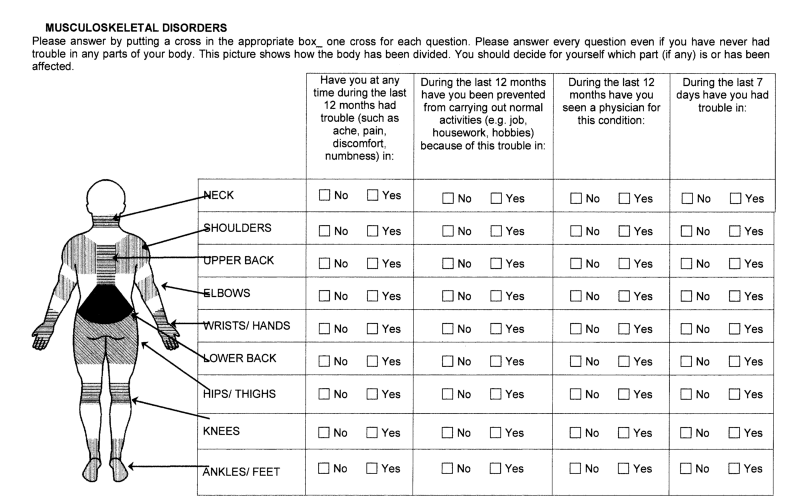
**

**Dziękujemy za wypełnienie ankiety**
